# An Engineered clMagR Tetramer with Enhanced Magnetism for Magnetic Manipulation

**DOI:** 10.3390/biom16040537

**Published:** 2026-04-03

**Authors:** Peng Zhang, Xiujuan Zhou, Shenting Zhang, Peilin Yang, Zhu-An Xu, Xin Zhang, Junfeng Wang, Tiantian Cai, Yuebin Zhang, Can Xie

**Affiliations:** 1 High Magnetic Field Laboratory, Hefei Institutes of Physical Science, Chinese Academy of Sciences, Hefei 230031, China; zhangpeng@mail.ustc.edu.cn (P.Z.); xinzhang@hmfl.ac.cn (X.Z.); junfeng@hmfl.ac.cn (J.W.); 2 Science Island Branch of Graduate School, University of Science and Technology of China, Hefei 230036, China; 3 Institute of Quantum Sensing, Zhejiang University, Hangzhou 310027, China; tiantiancai@zju.edu.cn; 4 Institute of Vegetables, Anhui Academy of Agricultural Sciences, Hefei 230001, China; xiujuan1994@mail.ustc.edu.cn; 5 Interdisciplinary Research Center for Biology and Chemistry, Liaoning Normal University, Dalian 116029, China; zhangyb@dicp.ac.cn; 6 State Key Laboratory of Membrane Biology, School of Life Sciences, Peking University, Beijing 100871, China; yangpeilin@pku.edu.cn; 7 School of Physics, Zhejiang University, Hangzhou 310058, China; zhuan@zju.edu.cn; 8 State Key Laboratory of Silicon and Advanced Semiconductor Materials & School of Physics, Zhejiang University, Hangzhou 310058, China; 9 Institute of Fundamental and Transdisciplinary Research, Zhejiang University, Hangzhou 310027, China

**Keywords:** biomagnetic manipulation, MagR, SDT-MagR, exceptional stability, enhanced magnetism

## Abstract

Biological manipulation via physical stimuli such as light and magnetism has become a central goal in modern biotechnology. Among these modalities, magnetic fields offer unique advantages, including deep tissue penetration and untethered interventions in living systems. An ideal platform for such a magnetogenetic toolkit would be a genetically encodable protein with tunable magnetic features under physiological conditions. However, the development of such tools has been hindered by the lack of robust and stable protein scaffolds with strong intrinsic magnetic properties. Inspired by animal magnetoreception in nature, here, we rationally designed and systematically screened single-chain variants of the magnetoreceptor MagR. Through nine iterative rounds of design and experimental validation, we generated 25 constructs and ultimately identified a stable single-chain-dimer-based-tetramer, SDT-MagR, as the optimal magnetic molecular platform. This engineered protein exhibits exceptional structural stability and state-dependent magnetic behavior, showing ferrimagnetic-like characteristics in the solid state and paramagnetic behavior in solution. With enhanced magnetic susceptibility, purified SDT-MagR can be directly attracted by a magnet in vitro, establishing it as a promising new platform for future biomagnetic manipulation and magnetogenetics applications.

## 1. Introduction

The precise modulation of biomolecular activity, cellular states, and even animal behavior through physical stimuli such as light and magnetism has long been a scientific aspiration and a persistent pursuit for biotechnology. Compared to traditional chemical stimulation using agonists or inhibitors, optical and magnetic stimuli offer distinct advantages: low toxicity, rapid and precise action, high reversibility, and spatiotemporal resolution reaching the sub-millisecond to millisecond scale, enabling accurate targeting and modulation. Optical manipulation including optogenetics enables the manipulation of neuronal activity via light-sensitive membrane opsins, which has revolutionized neuroscience since its pioneering development in the 2000s [1,2]. However, its practical applications have been limited due to the requirement for invasive surgery and tethered light sources.

In contrast, magnetic fields offer unparalleled advantages as a three-dimensional, non-invasive physical stimulus with high tissue penetrance. The ability of magnetic fields to penetrate biological tissues allows for remote, non-invasive stimulation deep within organisms, such as into the brain’s interior [3]. Therefore, much as optogenetics has profoundly propelled neuroscience forward, biomagnetic control technology based on “magnetogenetics” holds immense potential to become another disruptive technology [4,5]. It promises to provide novel tools for fundamental research and new strategies for clinical therapeutics.

Compared to the remarkable progress that has been made in optogenetics, the much-anticipated development of “magnetogenetics” has been slow and challenging. In optogenetics, the molecular tools—photosensitive proteins—are well defined, and their phototransduction mechanisms are clearly understood, enabling precise optical control. In contrast, magnetogenetics lacks an equivalent molecular actuator. The main obstacle has been the absence of a genetically encodable protein with intrinsic and tunable magnetic properties that functions effectively under physiological conditions.

To date, most magnetogenetics approaches have relied on inorganic magnetic nanoparticles (MNP) or paramagnetic ferritin as magnetic modulation tools [3,6,7]. However, MNPs cannot be genetically encoded, thus it must be conjugated with other biological components (such as ion channel proteins) to function as a magnetic actuator, and ferritin must assemble into 24-mer and be loaded with ferric iron to exhibit weak paramagnetism [8,9], which imposes considerable limitations in practical applications. To overcome these obstacles, there is an urgent need to engineer a protein molecule with enhanced and tunable magnetic properties.

Identification of a highly magnetosensitive protein is crucial for developing next-generation magnetic actuators. Similar strategies have been utilized by nature. The fundamental mechanism of animal magnetoreception and navigation is achieved through the response of specific protein molecules to the geomagnetic field [10,11,12]. Thus, the molecular machinery used by migratory birds and pigeons undoubtedly offers the best possible solution.

Over more than a century of research, scientists have proposed various theories to explain how animals perceive the extremely weak geomagnetic field (~25–65 μT) for long-distance navigation. Currently, three different magnetic “receptors” have been proposed in three major theoretical models: the magnetite particles by biomineralization in magnetite model [13,14,15]; the cryptochrome protein (Cry) in radical pair model [11,16,17]; and the MagR/Cry protein complex in biocompass model [12,18]. Both Cry and MagR proteins can be genetically encoded. The Cry protein can detect the geomagnetic field, particularly magnetic inclination, through dynamics in electron spin states (singlet vs. triplet). However, it cannot discern the direction of the magnetic field, and it is currently unknown how organisms recognize changes in electron spin states and transmit this information to the nervous system. For biomagnetic control, this defect prevents us from using Cry directly as a molecular tool. Consistently, the magnetoresponsive protein Cry from the radical pair model of animal magnetoreception has not yet been reported for application in magnetogenetics. However, recent advances in protein engineering have begun to explore the direct manipulation of quantum spin states in designed protein scaffolds in a class of magneto-sensitive fluorescent proteins other than Cry. For instance, a 2026 study by Abrahams et al. demonstrated quantum spin resonance in engineered proteins including MagLOV, establishing a synthetic platform for multimodal sensing that leverages coherent spin dynamics [19]. While this innovative approach highlights the potential of utilizing spin physics in protein-based systems, it similarly faces the challenge of translating spin-state changes into a robust, genetically encodable output that can reliably modulate cellular function.

Among the three magnetoresponsive material bases identified in the fundamental research of animal magnetoreception, the MagR protein holds distinct advantages over magnetite and the Cry protein. As a highly conserved A-type iron–sulfur protein, MagR may play essential roles in animal magnetoreception, and its magnetic properties have been extensively studied. Over the past decades, experimental evidence has been accumulated [20,21]. Specifically, the magnetic sensitivity of MagR can be modulated by the valence states of its bound iron–sulfur clusters [20], and the presence of mononuclear iron [21], as well as its hierarchical protein assembly [22]. Furthermore, a research group in Japan, using small-angle X-ray scattering experiments, directly demonstrated that external magnetic fields induced the molecular assembly of MagR [23,24]. Practical applications based on MagR’s magnetic properties have also been widely explored with success. For example, MagR has been used as a magnetic protein tag to separate and purify target proteins with external magnetic fields [25,26,27]; A research group in South Korea applied MagR in a magnetic detection kit for SARS-CoV-2, achieving rapid and highly sensitive detection [28]; and MagR has also been applied as a contrast enhancer for magnetic resonance imaging [29,30]. Additionally, the MagR/Cry complex has been utilized as bionic magnetic sensors on a chip [31,32]. Most recently, in 2025, MagR was repurposed as a novel magnetogenetic actuator to enable remote neuromodulation of brain circuits [33]. More importantly, MagR can function as a single component that can be genetically encoded and expressed in any biological system.

The homology structural modeling of clMagR indicates that MagR tetramer serves as the basic building block of MagR polymer and likely represents the minimal functional magnetic unit, which has been confirmed by the rational design and characterization of single-chain MagR tetramer (SctMagR) [22]. However, the relatively weak magnetism and moderate stability of SctMagR limit its practical application, underscoring the need for more robust tetrameric designs with enhanced and tunable magnetic properties.

Here, we rationally designed and systematically generated a series of single-chain MagR variants to improve magnetic strength, stability, and self-assembly. Two complementary strategies including single-chain dimer and single-chain tetramer designs were employed across nine iterative rounds of design and validation, generating 25 distinct constructs. Each variant was then expressed, purified, and characterized. Several key biochemical and biophysical features including the iron–sulfur cluster binding, protein stability, oligomeric assembly, and magnetic property were used for screening and evaluation. Among these, one particular variant, SDT-MagR, designated as Single-chain-Dimer-based Tetramer MagR, was sorted out during this process, with remarkably prolonged protein stability, strong protein assembly capability and enhanced magnetism.

Magnetic characterization of SDT-MagR revealed distinct magnetic features, such as ferrimagnetism in the pellet-enriched (insoluble) form and paramagnetism in solution-phase, indicating state-dependent and tunable magnetism. This tunable magnetic property of SDT-MagR not only implies a magnetic regulatory mechanism which has not been revealed yet, but also establishes SDT-MagR as a molecular platform for future development of the next-generation of biomagnetic manipulation technologies, including magnetogenetics.

## 2. Materials and Methods

### 2.1. Protein Synthesis, Expression and Purification

Based on the previously reported clMagR protein sequence [12] and guided by an earlier single-chain tetrameric construct [22], we rationally designed 25 artificial single-chain MagR variants—comprising 5 single-chain dimers and 20 single-chain tetramers—by integrating computational modeling with established principles of protein design. The coding sequences of these 25 constructs were first optimized according to the codon usage bias of *E. coli* and subsequently synthesized and subcloned by Sangon Biotech (Shanghai, China) into the same expression vector used for clMagR.

For simultaneous batch comparison and screening, all single-chain MagR proteins were expressed in *E. coli* and purified under conditions identical to those used for clMagR. Each protein was N-terminally fused with a Strep-tag II affinity tag for purification purposes, and the expression vectors were designated as “pNS-”, which were engineered by modifying the pCold expression vector, equipped with a kanamycin resistance gene, and capable of both cold and chemical induction. Each plasmid was individually transformed into *E. coli* BL21 (DE3) strains for induced expression. Following selection on antibiotic plates (indicating successful transformation), *E. coli* cultures were initially inoculated into 30 mL of LB medium containing kanamycin (50 μg/mL) and grown at 220 rpm and 37 °C until an OD_600_ of approximately 0.6 was reached. The cultures were then scaled up by transferring 30 mL of culture into 700 mL of fresh, sterile LB medium supplemented with kanamycin under the same growth conditions until reaching an OD_600_ of around 0.6 again. Subsequently, cultures were pre-chilled for 1 h at 160 rpm and 16 °C before inducing protein expression with IPTG at a final concentration of 20 μM for 20–24 h. Cells expressing different proteins were harvested separately by centrifugation.

The collected bacterial pellets were resuspended in TBS (20 mM Tris, 150 mM NaCl, pH 8.0) and lysed using an ultrasonic homogenizer (Scientz, Ningbo, China). The lysate was then subjected to high-speed centrifugation (17,500 rpm, 4 °C) for 45 min to separate the supernatant from the pellet, with the former being used for subsequent protein purification. The supernatant was loaded onto a Strep-Tactin gravity flow column (IBA Lifesciences, Göttingen, Germany). The column was washed with S-W buffer (20 mM Tris, 500 mM NaCl, pH 8.0) until the absorbance at 280 nm (A280) of the eluted drops fell below 0.1, indicating complete washing. Target proteins were eluted using S-E buffer (20 mM Tris, 150 mM NaCl, 5 mM d-Desthiobiotin, pH 8.0). The purity of all proteins was assessed and analyzed by SDS-PAGE, and protein sizes were estimated using a prestained protein marker (Thermo Fisher Scientific, Waltham, MA, USA).

### 2.2. UV–Vis Absorption Analysis

UV–vis absorption spectroscopy is the most widely used and straightforward method for detecting iron–sulfur clusters. Following purification via Strep-Tactin affinity chromatography, all protein samples were normalized to equivalent concentrations (1 mg/mL) based on their absorbance at 280 nm. Subsequently, 200 µL of each protein solution was loaded onto a spectrophotometer (NanoDrop One^C^, Thermo Fisher Scientific, USA) to record the absorption spectrum from 300 to 600 nm at room temperature, with S-E buffer used as the blank reference.

### 2.3. Size Exclusion Chromatography and Electron Microscopy Observation

Size exclusion chromatography (SEC) is commonly employed to separate and purify proteins based on differences in their hydrodynamic radii, which correlate with molecular mass. For samples of high purity, SEC can also be used to assess oligomeric states. Following Strep-Tactin affinity purification, all protein samples were normalized to equivalent concentrations (2 mg/mL) based on their absorbance at 280 nm. Subsequently, 200 µL of each sample was subjected to SEC using an ÄKTA pure chromatography system (Cytiva, Marlborough, MA, USA) equipped with a Superose 6 Increase 10/300 column (Cytiva, USA) at a flow rate of 0.5 mL/min. The column and system were equilibrated with TBS buffer (20 mM Tris, 150 mM NaCl, pH 8.0). The oligomeric state of each protein was inferred from its elution profile monitored by absorbance at 280 nm (A_280_).

To further assess the assembly states of the purified proteins, we performed transmission electron microscopy (TEM) analysis on freshly purified clMagR and SDT-MagR. Following Strep-Tactin affinity chromatography, protein samples were adjusted to equivalent concentrations (0.02 mg/mL) based on their A_280_ absorbance and negatively stained on carbon-coated copper grids according to a previously reported protocol [12]. All the grids were imaged using a Talos F200X transmission electron microscope (Thermo Fisher Scientific, USA) operated at 120 kV.

### 2.4. Electron Paramagnetic Resonance Measurement

To further characterize the type and redox state of the iron–sulfur clusters bound to the protein, X-band (∼9.6 GHz) electron paramagnetic resonance (EPR) spectra were recorded for both oxidized and reduced protein samples using an EMXplus 10/12 spectrometer (Bruker BioSpin GmbH, Rheinstetten, Germany) equipped with an Oxford ESR-910 liquid helium cryostat.

All purification steps were carried out under aerobic conditions without the addition of reducing agents to the buffers; consequently, the protein eluted from the Strep-Tactin column was in the oxidized state. The reduced sample was prepared by adding sodium dithionite (Na_2_S_2_O_4_) to freshly purified protein to a final concentration of 10 mM. For EPR measurements, 50 µL of glycerol was added to 200 µL of protein solution (1 mM, either oxidized or reduced) to achieve a final glycerol concentration of ∼20% (*v*/*v*), ensuring cryoprotection. The mixture was thoroughly mixed and loaded into 4-mm outer diameter quartz EPR tubes, which were rapidly frozen in liquid nitrogen.

EPR spectra were acquired at multiple temperatures (10 K, 25 K, 45 K, and 60 K). Instrumental parameters were as follows: microwave frequency, 9.40 GHz; microwave power, 2 mW; modulation amplitude, 2 G; and scan time, 25.6 s.

### 2.5. Ferrozine Assay

Dissolved ferrous ions react with ferrozine to form an intensively purple complex, which exhibits a characteristic absorption peak at 562 nm [34]. Therefore, ferrozine can be utilized for the quantitative analysis of iron in protein solutions. After purification with a Strep-Tactin column, the protein was adjusted to an A280 concentration of 1 mg/mL for the determination of total iron content. Mix 20 μL of sample or standard with 80 μL of hydroxylamine hydrochloride (HAHCl, 10% (*w*/*v*) in 1 M HCl) in a 96-well plate, and incubate the mixture in the dark for 30 min at 37 °C. This step aims to fully reduce all iron within the proteins to ferrous ions. Subsequently, 100 μL of ferrozine (0.1% (*w*/*v*) in 50% (*w*/*v*) ammonium acetate) was added, followed by further incubation in the dark for 15 min at 37 °C. The absorbance at 562 nm was then measured and recorded using a microplate reader (Tecan Group Ltd., Männedorf, Switzerland). Ferric chloride at various concentrations (0–800 μmol/L) dissolved in 1 M HCl were processed following the same procedure as the samples to construct a standard curve. S-E buffer was used as a blank sample and treated similarly to account for background subtraction. Each protein sample was prepared in at least three replicates. Data analysis and histogram plotting were performed using GraphPad Prism v9.4.0.

### 2.6. Magnetic Property Measurement

A superconducting quantum interference device (SQUID) is an extremely sensitive magnetometer that detects and measures tiny magnetic fields by exploiting quantum interference effects in superconducting circuits. In this study, we employed a DC-SQUID magnetometry mode (MPMS3; Quantum Design, San Diego, CA, USA) to directly measure the magnetic properties of the samples. A custom-made sample holder compatible with the instrument was designed for containing small-volume (approximately 170 μL) liquid samples (Quantum Design, USA). For solid samples, an appropriate amount was loaded into the sample holder and the mass of the sample was determined by subtracting the weight of the empty holder. Liquid samples were prepared by pipetting 170 μL into the sample holder, which was then carefully sealed before measurement. All measurements were conducted at a temperature of 298 K with a scan range of ±100 Oe. The results for solid samples were obtained by subtracting the signal from the empty sample holder, whereas those for liquid samples were derived by subtracting the corresponding buffer signals. Data analysis and graph plotting were performed using OriginPro 2025.

### 2.7. Bacteria Staining and Fluorescence Imaging

Following induction of EGFP, clMagR–EGFP, and SDT–EGFP as described above, bacterial cells were harvested by centrifugation. The cell pellets were washed three times with ddH_2_O to remove residual culture medium, followed by a final centrifugation to remove excess ddH_2_O. In a darkened room, the pellets were resuspended in 100 µL of Hoechst 33258 (20 µg/mL in ddH_2_O) and incubated at room temperature in the dark for 20 min. After centrifugation to remove the staining solution, the pellets were resuspended in 100 µL of FM4–64 (20 µg/mL in DMSO) and incubated on ice in the dark for 3 min. Finally, 90 µL of the dye solution was removed by centrifugation, and the remaining cell suspension was gently resuspended by pipetting and spread onto glass slides for fluorescence imaging. All fluorescence images were acquired using a laser scanning confocal microscope (Leica Microsystems, Wetzlar, Germany).

### 2.8. Sucrose Density Gradient Centrifugation and Magnetic Attraction

Density gradient centrifugation separates biological components—such as organelles, viruses, nucleic acids, or proteins—based on differences in their sedimentation rates through a density gradient medium under centrifugal force [35,36,37]. In this study, we employed sucrose density gradient centrifugation to fractionate the ultrasonicated lysate of *E. coli* expressing SDT.

First, sucrose solutions at 60%, 70%, and 80% (*w*/*v*) were freshly prepared, fully dissolved, and pre-chilled at 4 °C for 30 min. Using a pipette, 10 mL of each solution was carefully layered in a centrifuge tube (compatible with a Beckman SW32 Ti swinging-bucket rotor) in descending order—from bottom to top: 80%, 70%, and 60% sucrose—to establish a step gradient. Two identical gradients were prepared: one for fractionation and SDS-PAGE analysis, and the other for subsequent magnetic attraction assays. Next, 5 mL of the clarified lysate from SDT-expressing *E. coli* (following sonication) was gently overlaid onto the top of each sucrose gradient. The tubes were then subjected to ultracentrifugation (Beckman Coulter, Brea, CA, USA) at 60,000× *g* for 2 h at 4 °C. After centrifugation, a distinct thin brownish-yellow band—enriched in SDT—was clearly visible at the interface between the 70% and 80% sucrose layers.

For analysis, one gradient was carefully fractionated from top to bottom, including the interfacial layers between sucrose steps, and each fraction was analyzed by SDS-PAGE. The second gradient was placed adjacent to the central region of a custom-assembled neodymium–iron–boron (NdFeB) permanent magnet, where the magnetic field strength is approximately 1 T. The tube was securely fixed in place with transparent tape and incubated undisturbed for 24 h to assess magnetic attraction of SDT. As a blank control, *E. coli* cells not expressing any exogenous protein were processed identically through the entire density gradient centrifugation and fractionation procedure.

## 3. Results

### 3.1. Design and Screening of Single-Chain MagR Variants

**Figure 1:**
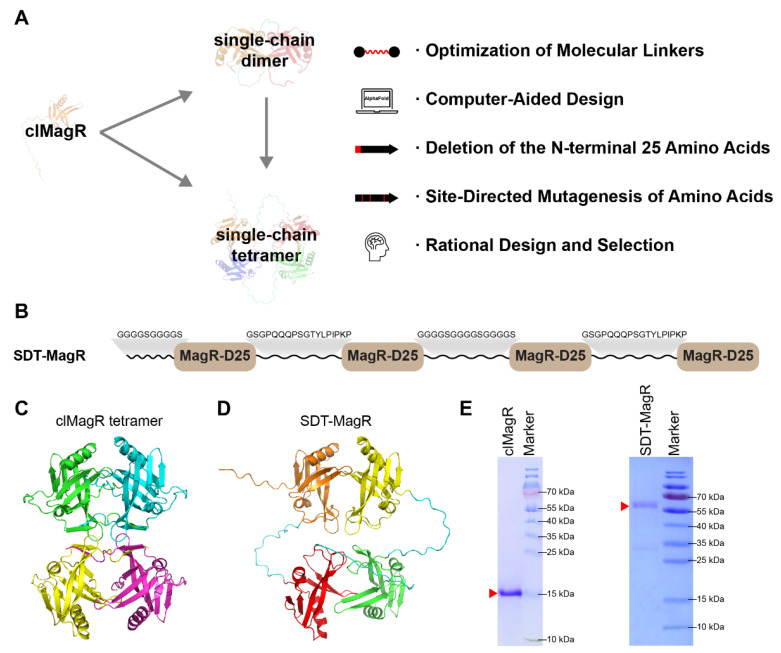
Design and structural characterization of single-chain MagR variants. (**A**) Schematic illustration of the rational design strategy for single-chain MagR constructs. Both single-chain dimers and single-chain tetramers were engineered based on the native MagR sequence; additionally, some single-chain tetramers were also derived from the single-chain dimer scaffold. The five key design principles guiding this process are summarized on the right. (**B**) Schematic representation of the single-chain tetramer SDT-MagR, comprising four N-terminally truncated MagR fragments (MagR-D25) linked by four flexible peptide linkers. (**C**) AlphaFold2-predicted homotetrameric structural model of clMagR. (**D**) AlphaFold2-predicted structural model of single-chain tetramer SDT-MagR. (**E**) SDS-PAGE analysis of purified clMagR and SDT-MagR proteins. Red arrows indicate the corresponding protein bands.

Extensive studies have been focused on the structural architecture of MagR’s hierarchical assemblies [12,18,20,22], which established the concept that the tetramer served as functional unit of MagR polymer. Thus, in this study, we aimed to stabilize the native tetrameric building block while enhancing its magnetic responsiveness. To this end, a library of rationally designed single-chain MagR constructs was generated, encompassing both single-chain dimers (SCDs) and single-chain tetramers (SCTs). Each design was guided by five principles: (i) optimization of the intramolecular linker length and flexibility; (ii) computational modeling and construction of both dimeric and tetrameric topology; (iii) inclusion or deletion of the N-terminal 25 amino acids to preserve the potential of MagR/Cry complex formation or to improve solubility of the MagR tetramer; (iv) site-directed mutagenesis at interfacial residues to strengthen hydrophobic packing; and (v) comparative biochemical screening based on Fe–S cluster incorporation, oligomerization state, and expression yield (Figure 1A, Supplementary Table S1).

Among the screened constructs, a single-chain-dimer-based tetramer, designated SDT-MagR, emerged as the optimal design after several rounds of design and rigorous screening (Figure 1B). Overall, SDT consists of four MagR-D25 units, and the first MagR-D25 is preceded by a (GGGGS)_2_ linker (GS linker [38,39]) to ensure proper folding by separating it from the N-terminal affinity tag used during protein purification. The linkers between the first and second, and third and fourth MagR-D25 units were computationally optimized, while the linker between the second and third MagR-D25 units (i.e., between two SCDs) was manually designed as a GS linker. Structural modeling using AlphaFold2 [40] predicted that SDT-MagR preserves the overall folding of native clMagR tetramers, with flexible linkers to enhance intramolecular stability a (Figure 1C,D). The protein was then expressed in *E. coli* and purified to homogeneity using a single-step Strep-tag affinity purification (Figure 1E), indicating that the artificial linkage did not impair protein folding.

### 3.2. SDT-MagR Preserves MagR Biochemical Features and Exhibits Enhanced Stability

**Figure 2:**
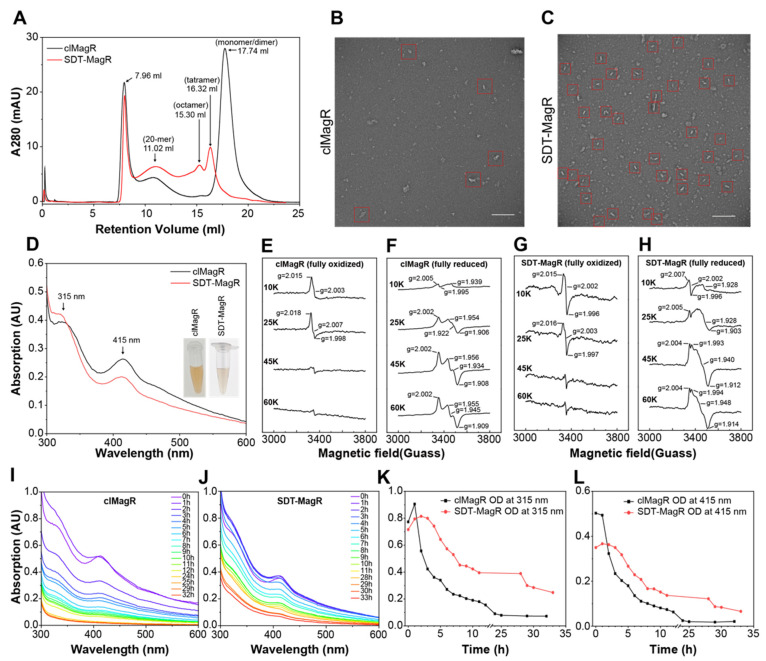
Comparative biochemical characterization of SDT and clMagR proteins. (**A**) Size exclusion chromatography (SEC) profiles of clMagR and SDT-MagR. Black arrows denote elution peaks and the inferred oligomeric states. (**B**,**C**) Transmission electron microscopy (TEM) images of freshly purified clMagR (**B**) and SDT-MagR (**C**) following negative staining. The red box highlights the putative rod-like 20-mer structure. Scale bar, 200 nm. (**D**) UV–vis absorption spectra of clMagR and SDT-MagR. Characteristic iron–sulfur cluster absorbance peaks are indicated by black arrows at 315 nm and 415 nm. The inset shows the color of the protein solutions. (**E**,**F**) X-band EPR spectra of oxidized (**E**) and dithionite-reduced (**F**) clMagR recorded at multiple temperatures (10 K, 25 K, 45 K, and 60 K). (**G**,**H**) X-band EPR spectra of oxidized (**G**) and dithionite-reduced (**H**) SDT-MagR recorded at multiple temperature conditions (10 K, 25 K, 45 K, and 60 K). (**I**,**J**) A time series of UV-visible absorption spectra of clMagR (**I**) and SDT-MagR (**J**) incubated at 37 °C. (**K**,**L**) Absorbance at 315 nm (**K**) and 415 nm (**L**) over time at 37 °C, derived from the spectra shown in (**I**,**J**). clMagR and SDT-MagR were assayed at comparable protein concentrations, enabling direct comparison of iron–sulfur cluster stability under identical conditions.

We next compared the biochemical and biophysical properties of SDT-MagR with those of native clMagR. Size exclusion chromatography (SEC) profiles revealed the peak corresponding to the 20-mer was significantly more prominent in SDT-MagR than in native clMagR, indicating a higher proportion of assembled species (Figure 2A). This observation was directly confirmed by TEM: in samples purified solely by Strep-affinity chromatography, SDT-MagR displayed a markedly higher abundance of rod-like structures compared to clMagR (Figure 2B,C).

UV–visible spectroscopy of both proteins revealed identical absorbance peaks at 315 nm and 415 nm (Figure 2D), characteristic of [2Fe–2S] and [3Fe–4S] clusters, respectively. The similar yellowish-brown coloration further confirmed proper incorporation of Fe–S cofactors in both SDT-MagR and clMagR. Electron paramagnetic resonance (EPR) spectra of both oxidized and reduced states of SDT-MagR and clMagR confirmed that SDT-MagR binds identical types of Fe–S clusters as clMagR (Figure 2G–J), validating that the engineered linkage does not perturb the magnetic cofactor environment.

To evaluate the protein stability, both proteins were incubated at 37 °C and monitored over time by UV–vis spectroscopy. Although Fe–S cluster absorbance gradually decreased in both proteins, SDT-MagR retained significantly higher absorbance at 315 nm and 415 nm (Figure 2I–L), indicating improved thermal and structural stability. Taken together, these data demonstrate that SDT-MagR not only preserves the key biochemical and structural features of clMagR but also exhibits enhanced stability and assembly capacity to form a functional rod-like 20-mer structure.

### 3.3. Distinct Subcellular Distribution and Ferrimagnetic Behavior of SDT-MagR in E. coli

**Figure 3:**
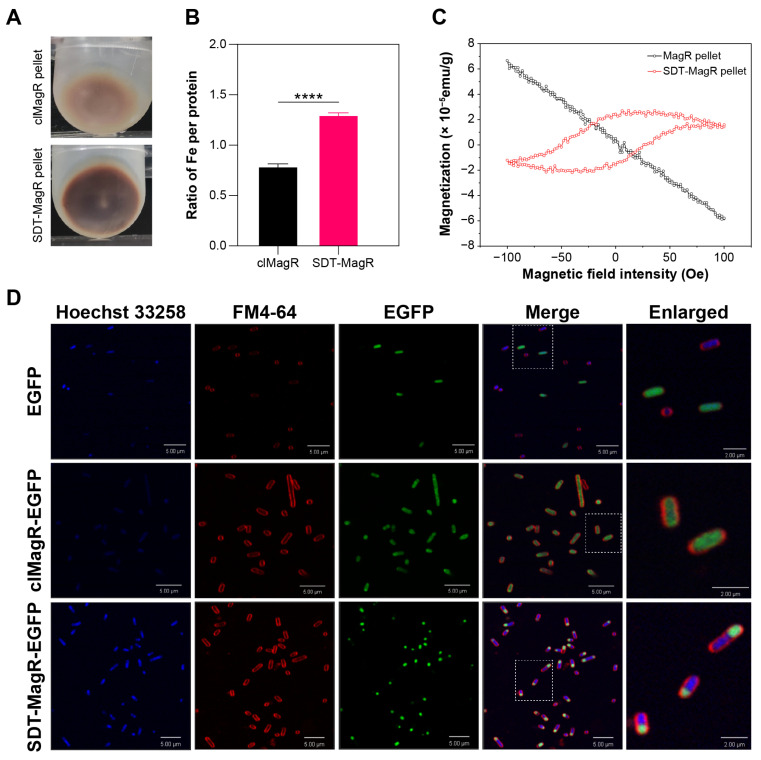
Differential expression patterns of SDT compared to clMagR in *E. coli*. (**A**) Pellets obtained after centrifugation of lysates from *E. coli* overexpressing clMagR and SDT-MagR. The SDT-MagR pellet appears visibly darker. One-way ANOVA, ****, *p* < 0.0001. (**B**) Total iron content measured by the ferrozine assay for purified clMagR and SDT-MagR at equivalent concentrations. (**C**) SQUID magnetometry measurements of pellets from lysates of *E. coli* overexpressing clMagR and SDT-MagR at 298 K. The SDT-MagR pellet exhibits a clear hysteresis loop, indicative of ferrimagnetic behavior, whereas the clMagR pellet shows diamagnetic characteristics with a magnetic susceptibility of approximately −6.2 × 10^−7^ cm^3^/g. The hysteresis loop yields coercive fields of +26 Oe and −50 Oe, with an exchange bias field (H_eb_) of 12 Oe and an exchange bias remanence (M_eb_) of 0.45 × 10^−5^ emu/g. (**D**) Confocal images of *E. coli* cells overexpressing EGFP, clMagR–EGFP, and SDT-MagR–EGFP, respectively. DNA and cell membranes were stained with Hoechst 33258 and FM4–64, respectively. Scale bar, 5 µm.

During protein purification, we noticed that bacterial pellets expressing SDT-MagR were markedly darker than those expressing clMagR (Figure 3A). Quantitative ferrozine assays confirmed that SDT-MagR contained substantially higher iron content (Figure 3B), suggesting enhanced iron and/or Fe–S clusters binding capacity and stability. To determine whether this difference led to enhanced magnetic properties, we analyzed the magnetic response of the crude lysate pellets by superconducting quantum interference device (SQUID) magnetometry. Strikingly, pellets expressing SDT-MagR displayed a ferrimagnetic hysteresis loop, whereas those expressing clMagR remained diamagnetic (Figure 3C). The observed asymmetry in the hysteresis loop—manifesting as unequal coercivities and remanences—is indicative of an internal bias field, likely arising from pinning of magnetic moments by stable iron–sulfur clusters within the aggregated SDT-MagR matrix (H_c_ = +26/−50 Oe; H_eb_ = 12 Oe; M_eb_ = 0.45 × 10^−5^ emu/g). This behavior resembles that reported in other cluster-based magnetic systems, where structural or chemical disorder induces unidirectional anisotropy [41,42].

The observed condensed pellet of SDT-MagR prompted us to investigate whether it exhibits a distinct subcellular expression pattern in *E. coli* compared to wild-type clMagR. To visualize subcellular localization, we fused both proteins with EGFP and examined *E. coli* cells under confocal microscopy. While clMagR–EGFP and EGFP alone showed diffuse cytoplasmic distribution, SDT-MagR–EGFP consistently formed a bright, single fluorescent focus near one cell pole (Figure 3D). This polar, aggregated expression pattern likely accounts for the high local protein density and the ferrimagnetic signal of SDT-MagR pellet observed in SQUID experiments. Together, these results suggest that the enhanced and ordered self-assembling (or ordered aggregation) and iron enrichment lead to the ferrimagnetic features, making SDT-MagR a potential magnetic tag for applications across different fields.

### 3.4. SDT-MagR Can Be Magnetically Attracted and Exhibits Tunable Magnetism

**Figure 4:**
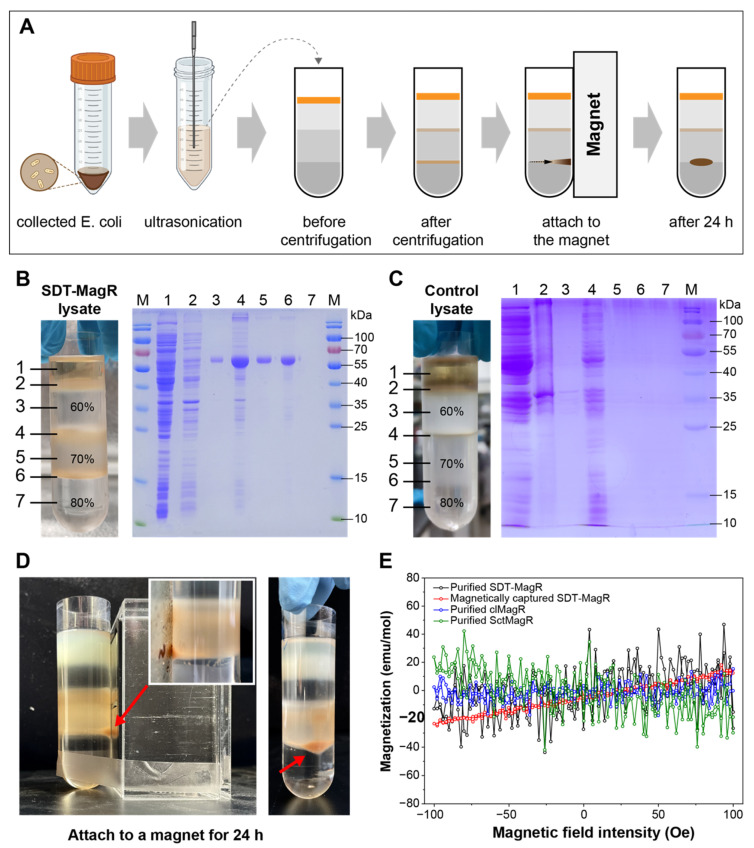
Density gradient centrifugation and magnetic capture of SDT. (**A**) Schematic overview of the experimental workflow for sucrose density gradient centrifugation and subsequent magnetic capture of SDT. (**B**) Representative image of the sucrose gradient following ultracentrifugation of lysate from *E. coli* overexpressing SDT-MagR, alongside SDS-PAGE analysis of fractions collected from each layer. (**C**) Equivalent sucrose density gradient centrifugation and SDS-PAGE analysis of fractions from lysate of control *E. coli* expressing no exogenous plasmid. (**D**) Photograph of the SDT-containing fraction after being attached to a 1 T permanent magnet for 24 h. The enriched brown material indicated by the red arrow represents magnetically captured SDT-MagR. (**E**) SQUID magnetometry measurements of purified clMagR, SctMagR, SDT protein, and the magnetically captured SDT sample (indicated by the red arrow in (**D**), showing differences in paramagnetic behavior among the samples. Molar magnetic susceptibilities were determined from linear fits and are approximately: SDT-MagR: 0.12 ± 0.0179; clMagR: 0.02 ± 0.00685; SctMagR: −0.17 ± 0.01498; magnetically captured SDT: 0.18 ± 0.002 (units: cm^3^/mol).

Considering the possible self-assembly and ordered aggregation of SDT-MagR as observed inside cells, sucrose density gradient centrifugation was applied to isolate SDT-MagR with different sizes, and magnetic enrichment was then performed after sucrose density gradient centrifugation (Figure 4A). After several rounds of optimization of various sucrose concentrations, the final sucrose density gradient (60%, 70%, and 80% *w*/*v*) was used to achieve the best separation of SDT-MagR. By this procedure, SDT-MagR concentrated primarily in the 70% layer and at the 70%/80% interface, appearing as a distinct brown band in the centrifugation tube, and the purity of isolated SDT-MagR was further confirmed by SDS–PAGE analysis (Figure 4B). Notably, no endogenous *E. coli* proteins or cellular components sedimented into the 70% sucrose layer under identical conditions (Figure 4C), confirming the specificity of SDT-MagR purification and also indicating the high density of SDT-MagR protein particles probably due to protein assembly and ordered aggregation.

When the centrifugation tube was placed adjacent to a 1 T permanent magnet for 24 h at room temperature, the brown SDT-MagR protein material visibly migrated toward the magnet-facing side (Figure 4D), demonstrating direct magnetic attraction.

The magnetically attracted SDT-MagR was collected for superconducting quantum interference device (SQUID) magnetometry, and clMagR, Sct-MagR and purified (but not magnetically attracted) SDT-MagR protein were used as controls. The SQUID data revealed that magnetically enriched SDT-MagR exhibits paramagnetic behavior and has the highest molar magnetic susceptibilities (0.18 ± 0.002 cm^3^/mol), which is about 9-folds enhanced compared with native clMagR protein (0.02 ± 0.00685 cm^3^/mol), with an uncertainty of roughly ±3.1, and also higher than the purified (but not magnetically attracted) SDT-MagR protein (0.12 ± 0.00179 cm^3^/mol) (Figure 4E). It is also important to point out that the previous version Sct-MagR protein showed slightly diamagnetic feature with molar magnetic susceptibilities measured as −0.17 ± 0.01498 cm^3^/mol (Figure 4E). Although the paramagnetic property of SDT-MagR protein is in contrast with the ferrimagnetic signal observed earlier in the crude pellet (which contained densely packed, solid-phase SDT aggregates), the differences in magnetic property could be due to the differences in molecular state: aggregated SDT-MagR in solid-phase pellets might have higher concentration and higher-order assembly, supporting long-range magnetic ordering, while dispersed SDT-MagR in solution behaves as a paramagnet due to decreased ordering of protein assembly and thermal fluctuations.

In all, these findings demonstrate that SDT-MagR not only preserves the biochemical and biophysical features including iron–sulfur cluster binding, self-assembly, and magnetic properties, but also improves these features with increased iron–sulfur cluster binding stability, better self-assembly, and enhanced and tunable magnetic susceptibilities. These improvements suggested that SDT-MagR, compared with native clMagR and the previous tetramer design Sct-MagR [22], is more suitable for use as a molecular tool for magnetogenetics and biological magnetic modulation.

## 4. Discussion

The magnetic properties of clMagR have long remained enigmatic, with the origin of its intrinsic magnetism unresolved due to the small number of iron atoms per protein unit [43]. Through rational protein engineering, we demonstrate here that enhancing MagR structural stability and promoting oligomerization can substantially amplify its magnetic properties. The engineered SDT-MagR, composed of four covalently linked MagR subunits, seems to retain the Fe–S cluster binding capacity and rod-like architecture of clMagR polymerization, while exhibiting significantly enhanced thermal stability and magnetic susceptibility.

Our findings suggest that the tetramer represents the minimal magnetic unit of MagR and that its higher-order assembly is crucial for magnetic ordering. The observation that SDT-MagR transitions between ferrimagnetic and paramagnetic states depending on its polymerization and ordering indicates a dynamic magnetism modulated by protein clustering and environmental context. Though the magnetism of SDT-MagR does not directly demonstrate the biological relevance of MagR as a compass protein, this behavior may indicate that a similar intrinsic mechanism could exist in vivo, whereby local protein density or redox state governs magnetic responsiveness during geomagnetic sensing.

To expand SDT-MagR as a molecular tool for biomagnetic manipulation, it is important to compare it with currently available approaches in magnetogenetics. Existing strategies broadly fall into two categories: exogenous MNPs and genetically encoded magnetic proteins such as ferritin and its variants. MNPs offer high magnetic susceptibility and well-established surface functionalization, but exogenous delivery is required due to their non-genetic encodability necessitates, limiting their use in long-term or in vivo studies. In contrast, genetically encoded ferritin allows targeted expression in specific cell populations, however, it must assemble into a 24-mer to function as a magnetic unit. In this context, SDT-MagR offers distinct advantages. First, as a genetically encodable single-protein construct, it enables cell-type-specific expression without exogenous particle delivery, circumventing issues of uptake heterogeneity and toxicity. Second, its engineered tetrameric architecture confers significantly enhanced magnetic susceptibility, achieving visible attraction to a permanent magnet, which is a property that has never been reported for ferritin or wild-type MagR under comparable conditions. Third, the covalently linked tetramer design improves thermal stability, facilitating handling and long-term function both in vivo and in vitro. Fourth, the dynamic, state-dependent magnetic switching (ferrimagnetic vs. paramagnetic) presents an opportunity for potentially reversible magnetic actuation.

From the perspective of potential applications, SDT-MagR represents a genetically encodable magnetic protein with tangible macroscopic magnetism, capable of being visibly attracted by a permanent magnet. This property establishes SDT-MagR as a promising molecular platform for biomagnetic manipulation, potentially enabling applications in protein separation, cellular control, and magnetogenetics.

It is worth pointing out that this study focused on the molecular design, construction, and biophysical characterization of SDT-MagR as a proof-of-concept tool for biological magnetic manipulation, as well as establishing a molecular foundation for mechanistic understanding of MagR magnetism. While SDT-MagR exhibits all essential properties for potential magnetogenetic applications, demonstrating such applications requires complex in vivo validation beyond the current scope. We anticipate that this work will provide a solid molecular foundation and stimulate future investigations to explore functional implementations of SDT-MagR across diverse biological contexts. Future studies will be required to couple SDT-MagR with biological effectors such as ion channels or signaling molecules to achieve magnetic control of cellular processes.

In summary, our study provides direct experimental evidence that engineered structural reinforcement and controlled oligomerization can yield a stable magnetic protein actuator. SDT-MagR not only offers a mechanistic model for elucidating the origin of MagR magnetism but also lays the groundwork for developing tunable magnetic tools for remote and noninvasive biological modulation.

## 5. Conclusions

In this study, we systematically designed a series of single-chain MagR variants based on the protein sequence of clMagR. Following prokaryotic expression, affinity purification, and comprehensive biochemical and biophysical characterization, we identified SDT-MagR, a single-chain tetrameric MagR variant with enhanced protein assembly capability, improved structural stability, and superior magnetic properties. Notably, SDT-MagR isolated via sucrose density gradient centrifugation demonstrated clear and robust responsiveness to external magnetic fields, as evidenced by its attraction to a permanent magnet. These features collectively indicate that SDT-MagR may serve as a highly promising next-generation molecular tool for biomagnetic manipulation technologies including magnetogenetics.

## Data Availability

Data is contained within the article or Supplementary Materials.

## Supplementary Materials

The following supporting information can be downloaded at: https://www.mdpi.com/article/10.3390/biom16040537/s1, Table S1: Sequence information of all designed single-chain MagR variants; Figure S1: Representative SDS–PAGE analysis of affinity-purified 25 single-chain MagR variants (corresponding to Table S1), showing the purity of each construct; Figure S2: Representative UV–vis absorption spectra of wild type MagR (WT-MagR) and 25 single-chain MagR variants (corresponding to Table S1); Figure S3: Representative size exclusion chromatography (SEC) profiles of wild type MagR (WT-MagR) and 25 single-chain MagR variants (corresponding to Table S1).
