# Supplementary material for: An Engineered clMagR Tetramer with Enhanced Magnetism for Magnetic Manipulation
Source: Biomolecules. 2026 Apr 3;16(4):537. doi: 10.3390/biom16040537 (PMC13113634; doi:10.3390/biom16040537)
Supplement: Supplementary file 1 [file biomolecules-16-00537-s001.zip › biomolecules-4212520-supplementary.pdf]

# *Supplementary Material*

## An Engineered clMagR Tetramer with Enhanced Magnetism for Magnetic Manipulation

Peng Zhang, Xiujuan Zhou, Shenting Zhang, Peilin Yang, Zhu-An Xu, Xin Zhang, Junfeng Wang, Tiantian Cai, Yuebin Zhang, Can Xie

**Table S1.** Sequence information of all designed single-chain MagR variants

| No. | Name | Simplified structure | Amino Acid Sequence |
|---|---|---|---|
| 1 | SctMagR | (GGGGS)$_2$-**(MagR-D25)**-(GGGGS)$_2$-**(MagR-D25)**-(GGGGS)$_3$-**(MagR-D25)**-(GGGGS)$_2$-**(MagR-D25)** | GGGGSGGGGSAALTLTPSAVQKIKELLKDKPEHVGVKVGVRTRGCNGLSYTLEYTKSKGDSDEEVVQDGVRVFIEKKAQLTLLGTEMDYVEDKLSSEFVFNNPNIKGTCGCGESFNIGGGGSGGGGSAALTLTPSAVQKIKELLKDKPEHVGVKVGVRTRGCNGLSYTLEYTKSKGDSDEEVVQDGVRVFIEKKAQLTLLGTEMDYVEDKLSSEFVFNNPNIKGTCGCGESFNIGGGGSGGGGSGGGGSAALTLTPSAVQKIKELLKDKPEHVGVKVGVRTRGCNGLSYTLEYTKSKGDSDEEVVQDGVRVFIEKKAQLTLLGTEMDYVEDKLSSEFVFNNPNIKGTCGCGESFNIGGGGSGGGGSAALTLTPSAVQKIKELLKDKPEHVGVKVGVRTRGCNGLSYTLEYTKSKGDSDEEVVQDGVRVFIEKKAQLTLLGTEMDYVEDKLSSEFVFNNPNIKGTCGCGESFNI* |
| 2 | E1-SCT | (GGGGS)$_2$-**(MagR-D25)**-(GGGGS)$_3$-**(MagR-D25)**-(GGGGS)$_3$-**(MagR-D25)**-(GGGGS)$_2$-**(MagR-D25)** | GGGGSGGGGSAALTLTPSAVQKIKELLKDKPEHVGVKVGVRTRGCNGLSYTLEYTKSKGDSDEEVVQDGVRVFIEKKAQLTLLGTEMDYVEDKLSSEFVFNNPNIKGTCGCGESFNIGGGGSGGGGSGGGGSAALTLTPSAVQKIKELLKDKPEHVGVKVGVRTRGCNGLSYTLEYTKSKGDSDEEVVQDGVRVFIEKKAQLTLLGTEMDYVEDKLSSEFVFNNPNIKGTCGCGESFNIGGGGSGGGGSGGGGSAALTLTPSAVQKIKELLKDKPEHVGVKVGVRTRGCNGLSYTLEYTKSKGDSDEEVVQDGVRVFIEKKAQLTLLGTEMDYVEDKLSSEFVFNNPNIKGTCGCGESFNIGGGGSGGGGSAALTLTPSAVQKIKELLKDKPEHVGVKVGVRTRGCNGLSYTLEYTKSKGDSDEEVVQDGVRVFIEKKAQLTLLGTEMDYVEDKLSSEFVFNNPNIKGTCGCGESFNI* |

| | | | |
|---|---|---|---|
| 3 | E2-SCT | $(GGGGS)_2$-**(MagR-D25)**-$(GGGGS)_2$-**(MagR-D25)**-$(GGGGS)_4$-**(MagR-D25)**-$(GGGGS)_2$-**(MagR-D25)** | GGGGSGGGGSAALTLTPSAVQKIKELLKDKPEHVGVKVGVRTRGCNGLSYTLEYTKSKGDSDEEVVQDGVRVFIEKKAQLTLLGTEMDYVEDKLSSEFVFNNPNIKGTCGCGESFNIGGGGSGGGGSAALTLTPSAVQKIKELLKDKPEHVGVKVGVRTRGCNGLSYTLEYTKSKGDSDEEVVQDGVRVFIEKKAQLTLLGTEMDYVEDKLSSEFVFNNPNIKGTCGCGESFNIGGGGSGGGGSGGGGSGGGGSAALTLTPSAVQKIKELLKDKPEHVGVKVGVRTRGCNGLSYTLEYTKSKGDSDEEVVQDGVRVFIEKKAQLTLLGTEMDYVEDKLSSEFVFNNPNIKGTCGCGESFNIGGGGSGGGGSAALTLTPSAVQKIKELLKDKPEHVGVKVGVRTRGCNGLSYTLEYTKSKGDSDEEVVQDGVRVFIEKKAQLTLLGTEMDYVEDKLSSEFVFNNPNIKGTCGCGESFNI* |
| 4 | E3-SCT | $(GGGGS)_2$-**(MagR-D25)**-$(GGGGS)_2$-**(MagR-D25)**-$(GGGGS)_3$-**(MagR-D25)**-$(GGGGS)_3$-**(MagR-D25)** | GGGGSGGGGSAALTLTPSAVQKIKELLKDKPEHVGVKVGVRTRGCNGLSYTLEYTKSKGDSDEEVVQDGVRVFIEKKAQLTLLGTEMDYVEDKLSSEFVFNNPNIKGTCGCGESFNIGGGGSGGGGSAALTLTPSAVQKIKELLKDKPEHVGVKVGVRTRGCNGLSYTLEYTKSKGDSDEEVVQDGVRVFIEKKAQLTLLGTEMDYVEDKLSSEFVFNNPNIKGTCGCGESFNIGGGGSGGGGSGGGGSAALTLTPSAVQKIKELLKDKPEHVGVKVGVRTRGCNGLSYTLEYTKSKGDSDEEVVQDGVRVFIEKKAQLTLLGTEMDYVEDKLSSEFVFNNPNIKGTCGCGESFNIGGGGSGGGGSGGGGSAALTLTPSAVQKIKELLKDKPEHVGVKVGVRTRGCNGLSYTLEYTKSKGDSDEEVVQDGVRVFIEKKAQLTLLGTEMDYVEDKLSSEFVFNNPNIKGTCGCGESFNI* |
| 5 | EA-SCT | $(GGGGS)_2$-**(MagR-D25)**-$(GGGGS)_3$-**(MagR-D25)**-$(GGGGS)_4$-**(MagR-D25)**-$(GGGGS)_3$-**(MagR-D25)** | GGGGSGGGGSAALTLTPSAVQKIKELLKDKPEHVGVKVGVRTRGCNGLSYTLEYTKSKGDSDEEVVQDGVRVFIEKKAQLTLLGTEMDYVEDKLSSEFVFNNPNIKGTCGCGESFNIGGGGSGGGGSGGGGSAALTLTPSAVQKIKELLKDKPEHVGVKVGVRTRGCNGLSYTLEYTKSKGDSDEEVVQDGVRVFIEKKAQLTLLGTEMDYVEDKLSSEFVFNNPNIKGTCGCGESFNIGGGGSGGGGSGGGGSGGGGSAALTLTPSAVQKIKELLKDKPEHVGVKVGVRTRGCNGLSYTLEYTKSKGDSDEEVVQDGVRVFIEKKAQLTLLGTEMDYVEDKLSSEFVFNNPNIKGTCGCGESFNIGGGGSGGGGSGGGGSAALTLTPSAVQKIKELLKDKPEHVGVKVGVRTRGCNGLSYTLEYTKSKGDSDEEVVQDGVRVFIEKKAQLTLLGTEMDYVEDKLSSEFVFNNPNI |

| | | | |
|---|---|---|---|
| | | | KGTCGCGESFNI* |
| 6 | SCD | (GGGGS)$_2$-**(MagR-D25)-**(GSGPQQQPSGTYLPIPKP)-**(MagR-D25)** | GGGGSGGGGSAALTLTPSAVQKIKELLKDKPEHVGVKVGVRTRGCNGLSYTLEYTKSKGDSDEEVVQDGVRVFIEKKAQLTLLGTEMDYVEDKLSSEFVFNNPNIKGTCGCGESFNIGSGPQQQPSGTYLPIPKPAALTLTPSAVQKIKELLKDKPEHVGVKVGVRTRGCNGLSYTLEYTKSKGDSDEEVVQDGVRVFIEKKAQLTLLGTEMDYVEDKLSSEFVFNNPNIKGTCGCGESFNI* |
| 7 | nSCT | (GGGGS)$_2$-**(MagR-D25)-**(GSGPQQQPSGTYLPIPKP)-**(MagR-D25)-**(AGDRTGPLGEQKPLEDFQ)**-(MagR-D25)-**(GSGPQQQPSGTYLPIPKP)-**(MagR-D25)** | GGGGSGGGGSAALTLTPSAVQKIKELLKDKPEHVGVKVGVRTRGCNGLSYTLEYTKSKGDSDEEVVQDGVRVFIEKKAQLTLLGTEMDYVEDKLSSEFVFNNPNIKGTCGCGESFNIGSGPQQQPSGTYLPIPKPAALTLTPSAVQKIKELLKDKPEHVGVKVGVRTRGCNGLSYTLEYTKSKGDSDEEVVQDGVRVFIEKKAQLTLLGTEMDYVEDKLSSEFVFNNPNIKGTCGCGESFNIAGDRTGPLGEQKPLEDFQAALTLTPSAVQKIKELLKDKPEHVGVKVGVRTRGCNGLSYTLEYTKSKGDSDEEVVQDGVRVFIEKKAQLTLLGTEMDYVEDKLSSEFVFNNPNIKGTCGCGESFNIGSGPQQQPSGTYLPIPKPAALTLTPSAVQKIKELLKDKPEHVGVKVGVRTRGCNGLSYTLEYTKSKGDSDEEVVQDGVRVFIEKKAQLTLLGTEMDYVEDKLSSEFVFNNPNIKGTCGCGESFNI* |
| 8 | S1F | **(MagR-FL)-**(GGGGS)$_2$-**(MagR-D25)-**(GGGGS)$_3$-**(MagR-D25)-**(GGGGS)$_2$-**(MagR-D25)** | MASSASSVVRATVRAVSKRKIQATRAALTLTPSAVQKIKELLKDKPEHVGVKVGVRTRGCNGLSYTLEYTKSKGDSDEEVVQDGVRVFIEKKAQLTLLGTEMDYVEDKLSSEFVFNNPNIKGTCGCGESFNIGGGGSGGGGSAALTLTPSAVQKIKELLKDKPEHVGVKVGVRTRGCNGLSYTLEYTKSKGDSDEEVVQDGVRVFIEKKAQLTLLGTEMDYVEDKLSSEFVFNNPNIKGTCGCGESFNIGGGGSGGGGSGGGGSAALTLTPSAVQKIKELLKDKPEHVGVKVGVRTRGCNGLSYTLEYTKSKGDSDEEVVQDGVRVFIEKKAQLTLLGTEMDYVEDKLSSEFVFNNPNIKGTCGCGESFNIGGGGSGGGGSAALTLTPSAVQKIKELLKDKPEHVGVKVGVRTRGCNGLSYTLEYTKSKGDSDEEVVQDGVRVFI |

| | | | |
|---|---|---|---|
| | | | EKKAQLTLLGTEMDYVEDKLSSEFVFNNPNIKGTCGCGESFNI* |
| 9 | S2F | **(MagR-FL)**-(GGGGS)$_2$-**(MagR-D25)**-(GGGGS)$_1$-**(MagR-FL)**-(GGGGS)$_2$-**(MagR-D25)** | MASSASSVVRATVRAVSKRKIQATRAALTLTPSAVQKIKELLKDKPEHVGVKVGVRTRGCNGLSYTLEYTKSKGDSDEEVVQDGVRVFIEKKAQLTLLGTEMDYVEDKLSSEFVFNNPNIKGTCGCGESFNIGGGGSGGGGSAALTLTPSAVQKIKELLKDKPEHVGVKVGVRTRGCNGLSYTLEYTKSKGDSDEEVVQDGVRVFIEKKAQLTLLGTEMDYVEDKLSSEFVFNNPNIKGTCGCGESFNIGGGGSMASSASSVVRATVRAVSKRKIQATRAALTLTPSAVQKIKELLKDKPEHVGVKVGVRTRGCNGLSYTLEYTKSKGDSDEEVVQDGVRVFIEKKAQLTLLGTEMDYVEDKLSSEFVFNNPNIKGTCGCGESFNIGGGGSGGGGSAALTLTPSAVQKIKELLKDKPEHVGVKVGVRTRGCNGLSYTLEYTKSKGDSDEEVVQDGVRVFIEKKAQLTLLGTEMDYVEDKLSSEFVFNNPNIKGTCGCGESFNI* |
| 10 | E1F | **(MagR-FL)**-(GGGGS)$_3$-**(MagR-D25)**-(GGGGS)$_4$-**(MagR-D25)**-(GGGGS)$_3$-**(MagR-D25)** | MASSASSVVRATVRAVSKRKIQATRAALTLTPSAVQKIKELLKDKPEHVGVKVGVRTRGCNGLSYTLEYTKSKGDSDEEVVQDGVRVFIEKKAQLTLLGTEMDYVEDKLSSEFVFNNPNIKGTCGCGESFNIGGGGSGGGGSGGGGSAALTLTPSAVQKIKELLKDKPEHVGVKVGVRTRGCNGLSYTLEYTKSKGDSDEEVVQDGVRVFIEKKAQLTLLGTEMDYVEDKLSSEFVFNNPNIKGTCGCGESFNIGGGGSGGGGSGGGGSGGGGSAALTLTPSAVQKIKELLKDKPEHVGVKVGVRTRGCNGLSYTLEYTKSKGDSDEEVVQDGVRVFIEKKAQLTLLGTEMDYVEDKLSSEFVFNNPNIKGTCGCGESFNIGGGGSGGGGSGGGGSAALTLTPSAVQKIKELLKDKPEHVGVKVGVRTRGCNGLSYTLEYTKSKGDSDEEVVQDGVRVFIEKKAQLTLLGTEMDYVEDKLSSEFVFNNPNIKGTCGCGESFNI* |

| | | | |
|---|---|---|---|
| 11 | E2F | **(MagR-FL)-** (GGGGS)$_3$- **(MagR-D25)-** (GGGGS)$_4$- **(MagR-FL)-** (GGGGS)$_3$- **(MagR-D25)** | MASSASSVVRATVRAVSKRKIQATRAALTLTPSAVQKIKELLKDKPEHVGVKVGVRTRGCNGLSYTLEYTKSKGDSDEEVVQDGVRVFIEKKAQLTLLGTEMDYVEDKLSSEFVFNNPNIKGTCGCGESFNIGGGGSGGGGSGGGGSAALTLTPSAVQKIKELLKDKPEHVGVKVGVRTRGCNGLSYTLEYTKSKGDSDEEVVQDGVRVFIEKKAQLTLLGTEMDYVEDKLSSEFVFNNPNIKGTCGCGESFNIGGGGSGGGGSMASSASSVVRATVRAVSKRKIQATRAALTLTPSAVQKIKELLKDKPEHVGVKVGVRTRGCNGLSYTLEYTKSKGDSDEEVVQDGVRVFIEKKAQLTLLGTEMDYVEDKLSSEFVFNNPNIKGTCGCGESFNIGGGGSGGGGSGGGGSAALTLTPSAVQKIKELLKDKPEHVGVKVGVRTRGCNGLSYTLEYTKSKGDSDEEVVQDGVRVFIEKKAQLTLLGTEMDYVEDKLSSEFVFNNPNIKGTCGCGESFNI* |
| 12 | F2D | **(MagR-FL)-** (GGGGS)$_2$- **(MagR-D25)** | MASSASSVVRATVRAVSKRKIQATRAALTLTPSAVQKIKELLKDKPEHVGVKVGVRTRGCNGLSYTLEYTKSKGDSDEEVVQDGVRVFIEKKAQLTLLGTEMDYVEDKLSSEFVFNNPNIKGTCGCGESFNIGGGGSGGGGSAALTLTPSAVQKIKELLKDKPEHVGVKVGVRTRGCNGLSYTLEYTKSKGDSDEEVVQDGVRVFIEKKAQLTLLGTEMDYVEDKLSSEFVFNNPNIKGTCGCGESFNI* |
| 13 | F3D | **(MagR-FL)-** (GGGGS)$_3$- **(MagR-D25)** | MASSASSVVRATVRAVSKRKIQATRAALTLTPSAVQKIKELLKDKPEHVGVKVGVRTRGCNGLSYTLEYTKSKGDSDEEVVQDGVRVFIEKKAQLTLLGTEMDYVEDKLSSEFVFNNPNIKGTCGCGESFNIGGGGSGGGGSGGGGSAALTLTPSAVQKIKELLKDKPEHVGVKVGVRTRGCNGLSYTLEYTKSKGDSDEEVVQDGVRVFIEKKAQLTLLGTEMDYVEDKLSSEFVFNNPNIKGTCGCGESFNI* |
| 14 | D2D | (GGGGS)$_2$- **(MagR-D25)-** (GGGGS)$_2$- **(MagR-D25)** | GGGGSGGGGSAALTLTPSAVQKIKELLKDKPEHVGVKVGVRTRGCNGLSYTLEYTKSKGDSDEEVVQDGVRVFIEKKAQLTLLGTEMDYVEDKLSSEFVFNNPNIKGTCGCGESFNIGGGGSGGGGSAALTLTPSAVQKIKELLKDKPEHVGVKVGVRTRGCNGLSYTLEYTKSKGDSDEEVVQDGVRVFIEKKAQLTLLGTEMDYVEDKLSSEFVFNNPNIKGTCGCGESFNI* |
| 15 | FCD | **(MagR-FL)-** (GSGPQQQPS GTYLPIPKP)- **(MagR-D25)** | MASSASSVVRATVRAVSKRKIQATRAALTLTPSAVQKIKELLKDKPEHVGVKVGVRTRGCNGLSYTLEYTKSKGDSDEEVVQDGVRVFIEKKAQLTLLGTEMDYVEDKLSSEFVFNNPNIKGTCGCGESFNIGSGPQQQPSGTYLPIPKPAALTLTPSAVQKIKELLKDKPEHV |

| | | | |
|---|---|---|---|
| | | | GVKVGVRTRGCNGLSYTLEYTKSKGDSDEEVVQDGVRVFIEKKAQLTLLGTEMDYVEDKLSSEFVFNNPNIKGTCGCGESFNI* |
| 16 | SDT | (GGGGS)2-**(MagR-D25)**-(GSGPQQQPSGTYLPIPKP)-**(MagR-D25)**-(GGGGS)3-**(MagR-D25)**-(GSGPQQQPSGTYLPIPKP)-**(MagR-D25)** | GGGGSGGGGSAALTLTPSAVQKIKELLKDKPEHVGVKVGVRTRGCNGLSYTLEYTKSKGDSDEEVVQDGVRVFIEKKAQLTLLGTEMDYVEDKLSSEFVFNNPNIKGTCGCGESFNIGSGPQQQPSGTYLPIPKPAALTLTPSAVQKIKELLKDKPEHVGVKVGVRTRGCNGLSYTLEYTKSKGDSDEEVVQDGVRVFIEKKAQLTLLGTEMDYVEDKLSSEFVFNNPNIKGTCGCGESFNIGGGGSGGGGSGGGGSAALTLTPSAVQKIKELLKDKPEHVGVKVGVRTRGCNGLSYTLEYTKSKGDSDEEVVQDGVRVFIEKKAQLTLLGTEMDYVEDKLSSEFVFNNPNIKGTCGCGESFNIGSGPQQQPSGTYLPIPKPAALTLTPSAVQKIKELLKDKPEHVGVKVGVRTRGCNGLSYTLEYTKSKGDSDEEVVQDGVRVFIEKKAQLTLLGTEMDYVEDKLSSEFVFNNPNIKGTCGCGESFNI* |
| 17 | SDT-Q36E | (GGGGS)2-**(MagR$^{Q36E}$-D25)**-(GSGPQQQPSGTYLPIPKP)-**(MagR$^{Q36E}$-D25)**-(GGGGS)3-**(MagR$^{Q36E}$-D25)**-(GSGPQQQPSGTYLPIPKP)-**(MagR$^{Q36E}$-D25)** | GGGGSGGGGSAALTLTPSAVEKIKELLKDKPEHVGVKVGVRTRGCNGLSYTLEYTKSKGDSDEEVVQDGVRVFIEKKAQLTLLGTEMDYVEDKLSSEFVFNNPNIKGTCGCGESFNIGSGPQQQPSGTYLPIPKPAALTLTPSAVEKIKELLKDKPEHVGVKVGVRTRGCNGLSYTLEYTKSKGDSDEEVVQDGVRVFIEKKAQLTLLGTEMDYVEDKLSSEFVFNNPNIKGTCGCGESFNIGGGGSGGGGSGGGGSAALTLTPSAVEKIKELLKDKPEHVGVKVGVRTRGCNGLSYTLEYTKSKGDSDEEVVQDGVRVFIEKKAQLTLLGTEMDYVEDKLSSEFVFNNPNIKGTCGCGESFNIGSGPQQQPSGTYLPIPKPAALTLTPSAVEKIKELLKDKPEHVGVKVGVRTRGCNGLSYTLEYTKSKGDSDEEVVQDGVRVFIEKKAQLTLLGTEMDYVEDKLSSEFVFNNPNIKGTCGCGESFNI* |
| 18 | SDT-T70D | (GGGGS)2-**(MagR$^{T70D}$-D25)**-(GSGPQQQPSGTYLPIPKP)-**(MagR$^{T70D}$-D25)**-(GGGGS)3-**(MagR$^{T70D}$-D25)**- | GGGGSGGGGSAALTLTPSAVQKIKELLKDKPEHVGVKVGVRTRGCNGLSYTLEYDKSKGDSDEEVVQDGVRVFIEKKAQLTLLGTEMDYVEDKLSSEFVFNNPNIKGTCGCGESFNIGSGPQQQPSGTYLPIPKPAALTLTPSAVQKIKELLKDKPEHVGVKVGVRTRGCNGLSYTLEYDKSKGDSDEEVVQDGVRVFIEKKAQLTLLGTEMDYVEDKLSSEFVFNNPNIKGTCGCGESFNIGGGGSGGGGSGGGGSAALTLTPSAVQKIKELLKDKPEHVGVKVGVRTRGCNGLSYTLEYDKSKGDSDEEVVQDGVRVFIEKKAQLTLLGTEMDYVEDKL |

| | | | |
|---|---|---|---|
| | | (GSGPQQQPSGTYLPIPKP)- **(MagR$^{T70D}$-D25)** | SSEFVFNNPNIKGTCGCGESFNIGSGPQQQPSGTYLPIPKPAALTLTPSAVQKIKELLKDKPEHVGVKVGVRTRGCNGLSYTLEYDKSKGDSDEEVVQDGVRVFIEKKAQLTLLGTEMDYVEDKLSSEFVFNNPNIKGTCGCGESFNI* |
| 19 | SDT-Q36E/T70D | (GGGGS)$_2$- **(MagR$^{Q36E/T70D}$-D25)**- (GSGPQQQPSGTYLPIPKP)- **(MagR$^{Q36E/T70D}$-D25)**- (GGGGS)$_3$- **(MagR$^{Q36E/T70D}$-D25)**- (GSGPQQQPSGTYLPIPKP)- **(MagR$^{Q36E/T70D}$-D25)** | GGGGSGGGGSAALTLTPSAVEKIKELLKDKPEHVGVKVGVRTRGCNGLSYTLEYDKSKGDSDEEVVQDGVRVFIEKKAQLTLLGTEMDYVEDKLSSEFVFNNPNIKGTCGCGESFNIGSGPQQQPSGTYLPIPKPAALTLTPSAVEKIKELLKDKPEHVGVKVGVRTRGCNGLSYTLEYDKSKGDSDEEVVQDGVRVFIEKKAQLTLLGTEMDYVEDKLSSEFVFNNPNIKGTCGCGESFNIGGGGSGGGGSGGGGSAALTLTPSAVEKIKELLKDKPEHVGVKVGVRTRGCNGLSYTLEYDKSKGDSDEEVVQDGVRVFIEKKAQLTLLGTEMDYVEDKLSSEFVFNNPNIKGTCGCGESFNIGSGPQQQPSGTYLPIPKPAALTLTPSAVEKIKELLKDKPEHVGVKVGVRTRGCNGLSYTLEYDKSKGDSDEEVVQDGVRVFIEKKAQLTLLGTEMDYVEDKLSSEFVFNNPNIKGTCGCGESFNI* |
| 20 | SDT2.1 | **(MagR$^{Q36E}$-FL)**- (GSGPQQQPSGTYLPIPKP)- **(MagR$^{Q36E}$-D25)**- (GGGGS)$_3$- **(MagR$^{Q36E}$-D25)**- (GSGPQQQPSGTYLPIPKP)- **(MagR$^{Q36E}$-D25)** | MASSASSVVRATVRAVSKRKIQATRAALTLTPSAVEKIKELLKDKPEHVGVKVGVRTRGCNGLSYTLEYTKSKGDSDEEVVQDGVRVFIEKKAQLTLLGTEMDYVEDKLSSEFVFNNPNIKGTCGCGESFNIGSGPQQQPSGTYLPIPKPAALTLTPSAVEKIKELLKDKPEHVGVKVGVRTRGCNGLSYTLEYTKSKGDSDEEVVQDGVRVFIEKKAQLTLLGTEMDYVEDKLSSEFVFNNPNIKGTCGCGESFNIGGGGSGGGGSGGGGSAALTLTPSAVEKIKELLKDKPEHVGVKVGVRTRGCNGLSYTLEYTKSKGDSDEEVVQDGVRVFIEKKAQLTLLGTEMDYVEDKLSSEFVFNNPNIKGTCGCGESFNIGSGPQQQPSGTYLPIPKPAALTLTPSAVEKIKELLKDKPEHVGVKVGVRTRGCNGLSYTLEYTKSKGDSDEEVVQDGVRVFIEKKAQLTLLGTEMDYVEDKLSSEFVFNNPNIKGTCGCGESFNI* |

| | | | |
|---|---|---|---|
| 21 | SDT2.2 | **(MagR$^{Q36E}$-FL)-** (GSGPQQQPSGTYLPIPKP)- **(MagR$^{Q36E}$-D25)-** (GGGGS)$_1$- **(MagR$^{Q36E}$-FL)-** (GSGPQQQPSGTYLPIPKP)- **(MagR$^{Q36E}$-D25)** | MASSASSVVRATVRAVSKRKIQATRAALTLTPSAVEKIKELLKDKPEHVGVKVGVRTRGCNGLSYTLEYTKSKGDSDEEVVQDGVRVFIEKKAQLTLLGTEMDYVEDKLSSEFVFNNPNIKGTCGCGESFNIGSGPQQQPSGTYLPIPKPAALTLTPSAVEKIKELLKDKPEHVGVKVGVRTRGCNGLSYTLEYTKSKGDSDEEVVQDGVRVFIEKKAQLTLLGTEMDYVEDKLSSEFVFNNPNIKGTCGCGESFNIGGGGSMASSASSVVRATVRAVSKRKIQATRAALTLTPSAVEKIKELLKDKPEHVGVKVGVRTRGCNGLSYTLEYTKSKGDSDEEVVQDGVRVFIEKKAQLTLLGTEMDYVEDKLSSEFVFNNPNIKGTCGCGESFNIGSGPQQQPSGTYLPIPKPAALTLTPSAVEKIKELLKDKPEHVGVKVGVRTRGCNGLSYTLEYTKSKGDSDEEVVQDGVRVFIEKKAQLTLLGTEMDYVEDKLSSEFVFNNPNIKGTCGCGESFNI* |
| 22 | 2309A | (GGGGS)$_2$- **(MagR$^{L95P/K108F/N131S}$-D25)-** (GSGPQQQPSGTYLPIPKP)- **(MagR$^{L95P/K108F/N131S}$-D25)-** (GGGGS)$_3$- **(MagR$^{L95P/K108F/N131S}$-D25)-** (GSGPQQQPSGTYLPIPKP)- **(MagR$^{L95P/K108F/N131S}$-D25)** | GGGGSGGGGSAALTLTPSAVQKIKELLKDKPEHVGVKVGVRTRGCNGLSYTLEYTKSKGDSDEEVVQDGVRVFIEKKAQPTLLGTEMDYVEDFLSSEFVFNNPNIKGTCGCGESFSIGSGPQQQPSGTYLPIPKPAALTLTPSAVQKIKELLKDKPEHVGVKVGVRTRGCNGLSYTLEYTKSKGDSDEEVVQDGVRVFIEKKAQPTLLGTEMDYVEDFLSSEFVFNNPNIKGTCGCGESFSIGGGGSGGGGSGGGGSAALTLTPSAVQKIKELLKDKPEHVGVKVGVRTRGCNGLSYTLEYTKSKGDSDEEVVQDGVRVFIEKKAQPTLLGTEMDYVEDFLSSEFVFNNPNIKGTCGCGESFSIGSGPQQQPSGTYLPIPKPAALTLTPSAVQKIKELLKDKPEHVGVKVGVRTRGCNGLSYTLEYTKSKGDSDEEVVQDGVRVFIEKKAQPTLLGTEMDYVEDFLSSEFVFNNPNIKGTCGCGESFSI* |
| 23 | 2309B | **(MagR$^{L95P/K108F/N131S}$-FL)-** (GSGPQQQPSGTYLPIPKP)- **(MagR$^{L95P/K108F/N131S}$-D25)-** (GGGGS)$_1$- **(MagR$^{L95P/K108F/N131S}$-FL)-** (GSGPQQQPSGTYLPIPKP)- **(MagR$^{L95P/K108F/N131S}$-D25)** | MASSASSVVRATVRAVSKRKIQATRAALTLTPSAVQKIKELLKDKPEHVGVKVGVRTRGCNGLSYTLEYTKSKGDSDEEVVQDGVRVFIEKKAQPTLLGTEMDYVEDFLSSEFVFNNPNIKGTCGCGESFSIGSGPQQQPSGTYLPIPKPAALTLTPSAVQKIKELLKDKPEHVGVKVGVRTRGCNGLSYTLEYTKSKGDSDEEVVQDGVRVFIEKKAQPTLLGTEMDYVEDFLSSEFVFNNPNIKGTCGCGESFSIGGGGSMASSASSVVRATVRAVSKRKIQATRAALTLTPSAVQKIKELLKDKPEHVGVKVGVRTRGCNGLSYTLEYTKSKGDSDEEVVQDGVRVFIEKKAQPTLLGTEMDYVEDFLSSEFVFNNPNIKGTCGCGESFSIGSGPQQQPSGTYLPIPKPAALTLTPSAVQKIKELLKDKPEHVGVKVGVRTRGCN |

| | | | |
|---|---|---|---|
| | | | GLSYTLEYTKSKGDSDEEVVQDGVRVFIEKKAQP<br>TLLGTEMDYVEDFLSSEFVFNNPNIKGTCGCGESF<br>SI* |
| 24 | 2401A | **(MagR$^{Q36E}$-FL)**-(GGGGS)$_1$-**(MagR$^{Q36E}$-FL)**-(GGGGS)$_1$-**(MagR$^{Q36E}$-FL)**-(GGGGS)$_1$-**(MagR$^{Q36E}$-FL)** | MASSASSVVRATVRAVSKRKIQATRAALTLTPSAV<br>EKIKELLKDKPEHVGVKVGVRTRGCNGLSYTLEY<br>TKSKGDSDEEVVQDGVRVFIEKKAQLTLLGTEMD<br>YVEDKLSSEFVFNNPNIKGTCGCGESFNIGGGGS<br>MASSASSVVRATVRAVSKRKIQATRAALTLTPSAV<br>EKIKELLKDKPEHVGVKVGVRTRGCNGLSYTLEY<br>TKSKGDSDEEVVQDGVRVFIEKKAQLTLLGTEMD<br>YVEDKLSSEFVFNNPNIKGTCGCGESFNIGGGGS<br>MASSASSVVRATVRAVSKRKIQATRAALTLTPSAV<br>EKIKELLKDKPEHVGVKVGVRTRGCNGLSYTLEY<br>TKSKGDSDEEVVQDGVRVFIEKKAQLTLLGTEMD<br>YVEDKLSSEFVFNNPNIKGTCGCGESFNIGGGGS<br>MASSASSVVRATVRAVSKRKIQATRAALTLTPSAV<br>EKIKELLKDKPEHVGVKVGVRTRGCNGLSYTLEY<br>TKSKGDSDEEVVQDGVRVFIEKKAQLTLLGTEMD<br>YVEDKLSSEFVFNNPNIKGTCGCGESFNI* |
| 25 | 2401B | **(MagR$^{Q36E/L95P/K108F/N131S}$-FL)**-(GGGGS)$_1$-**(MagR$^{Q36E/L95P/K108F/N131S}$-FL)**-(GGGGS)$_1$-**(MagR$^{Q36E/L95P/K108F/N131S}$-FL)**-(GGGGS)$_1$-**(MagR$^{Q36E/L95P/K108F/N131S}$-FL)** | MASSASSVVRATVRAVSKRKIQATRAALTLTPSAV<br>EKIKELLKDKPEHVGVKVGVRTRGCNGLSYTLEY<br>TKSKGDSDEEVVQDGVRVFIEKKAQPTLLGTEMD<br>YVEDFLSSEFVFNNPNIKGTCGCGESFSIGGGGSM<br>ASSASSVVRATVRAVSKRKIQATRAALTLTPSAVE<br>KIKELLKDKPEHVGVKVGVRTRGCNGLSYTLEYT<br>KSKGDSDEEVVQDGVRVFIEKKAQPTLLGTEMD<br>YVEDFLSSEFVFNNPNIKGTCGCGESFSIGGGGSM<br>ASSASSVVRATVRAVSKRKIQATRAALTLTPSAVE<br>KIKELLKDKPEHVGVKVGVRTRGCNGLSYTLEYT<br>KSKGDSDEEVVQDGVRVFIEKKAQPTLLGTEMD<br>YVEDFLSSEFVFNNPNIKGTCGCGESFSIGGGGSM<br>ASSASSVVRATVRAVSKRKIQATRAALTLTPSAVE<br>KIKELLKDKPEHVGVKVGVRTRGCNGLSYTLEYT<br>KSKGDSDEEVVQDGVRVFIEKKAQPTLLGTEMD<br>YVEDFLSSEFVFNNPNIKGTCGCGESFSI* |

Note：

1.MagR-D25 refers to a truncated variant of MagR lacking the N-terminal 25 amino acid residues；

2.MagR-FL refers to full-length MagR;

3.The amino acid sequence shown in red denotes the engineered intermolecular linker.

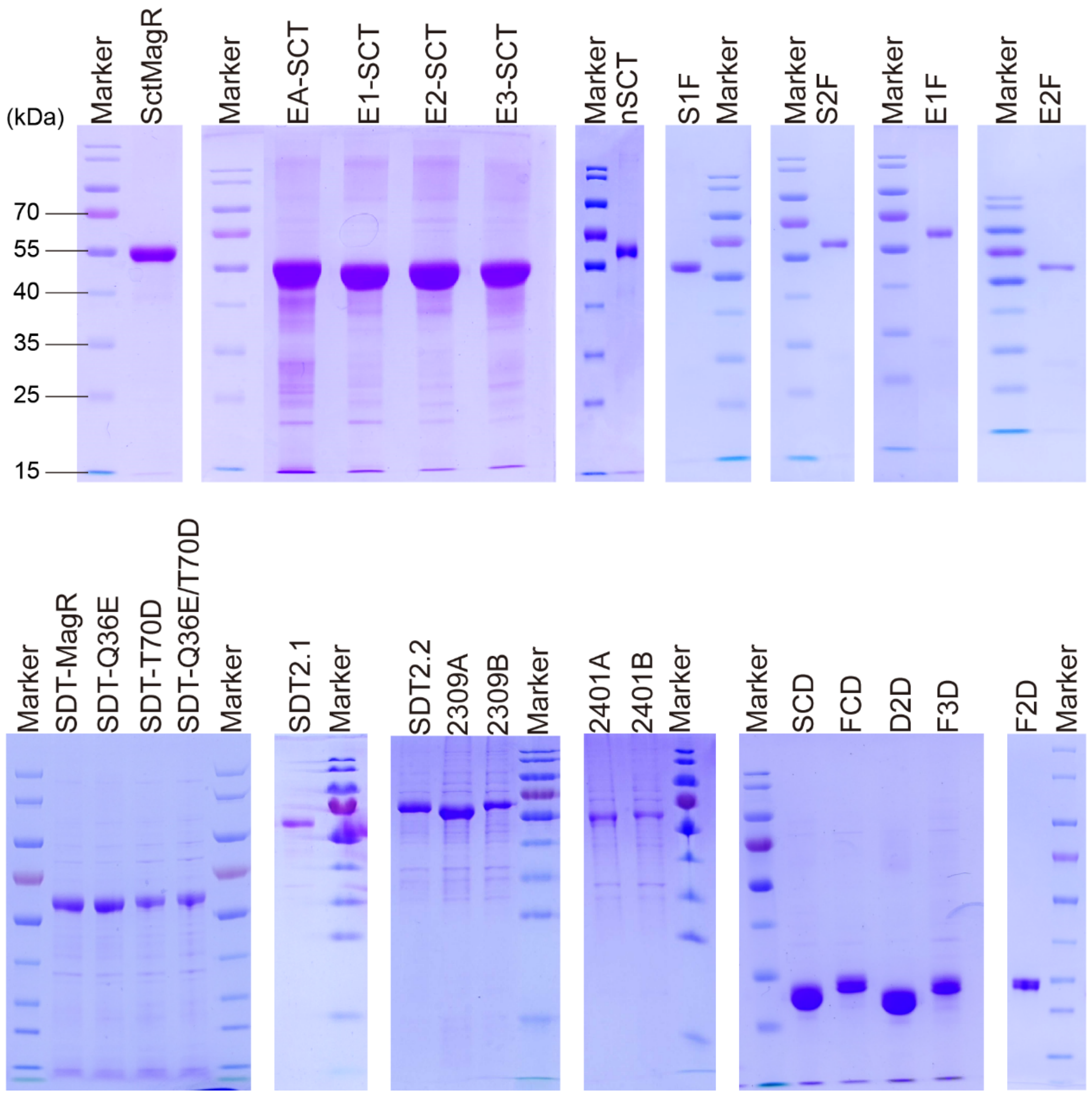


**Supplementary Figure 1.** Representative SDS–PAGE analysis of affinity-purified 25 single-chain MagR variants (corresponding to Table 1), showing the purity of each construct. The same prestained protein molecular weight marker was used throughout (Thermo Fisher Scientific, #26616).

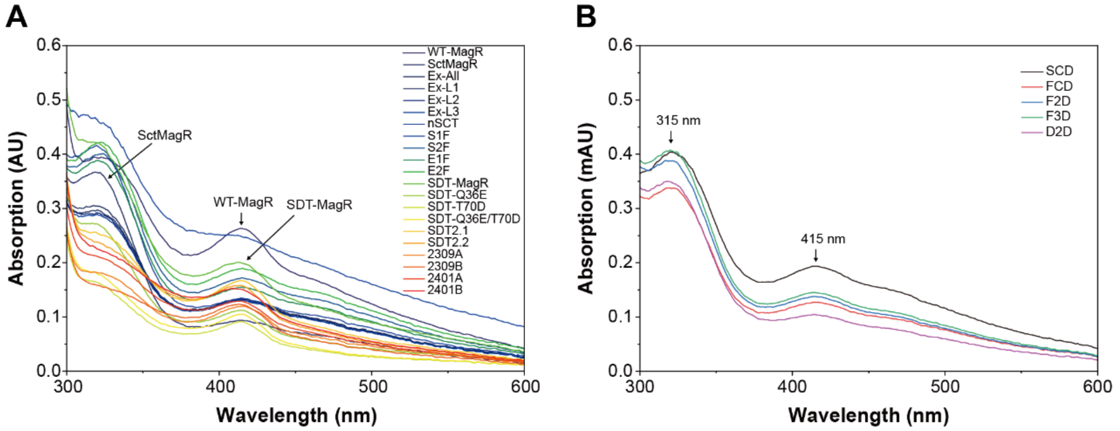


**Supplementary Figure 2.** Representative UV–vis absorption spectra of wild type MagR (WT-MagR) and 25 single-chain MagR variants (corresponding to Table 1). (a) Spectra of WT-MagR and 20 single-chain tetramers. The arrows indicate the UV-vis spectra of WT-MagR, SctMagR, and SDT-MagR, respectively. (b) Spectra of 5 single-chain dimers. All spectra were normalized to absorbance at 280 nm ($A_{280}$).

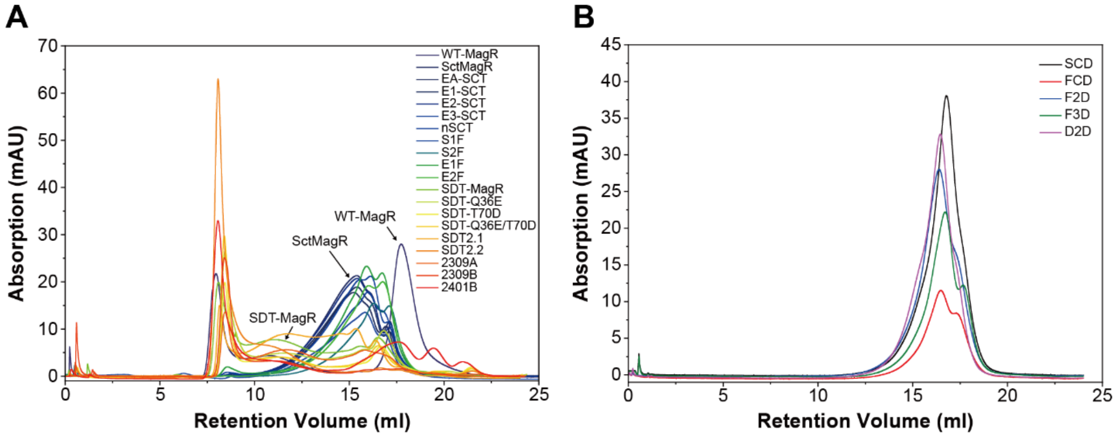


**Supplementary Figure 3.** Representative size-exclusion chromatography (SEC) profiles of wild type MagR (WT-MagR) and 25 single-chain MagR variants (corresponding to Table 1). (a) SEC profiles of WT-MagR and 19 single-chain tetramers (Construct 2401A was not analyzed by SEC due to insufficient expression yield). The arrows indicate the SEC profiles of WT-MagR, SctMagR, and SDT-MagR, respectively. (b) SEC profiles of 5 single-chain dimers. All runs were performed on a Superose 6 Increase 10/300 column (Cytiva, USA).
